# Application of Internet of Medical/Health Things to Decentralized Clinical Trials: Development Status and Regulatory Considerations

**DOI:** 10.3389/fmed.2022.903188

**Published:** 2022-06-06

**Authors:** Takahiro Sato, Hikaru Ishimaru, Takuya Takata, Hajime Sasaki, Mayumi Shikano

**Affiliations:** ^1^Astellas Pharma Inc., Tokyo, Japan; ^2^Department of Pharmaceutical Sciences, Tokyo University of Science, Tokyo, Japan; ^3^Institute for Future Initiatives, The University of Tokyo, Tokyo, Japan

**Keywords:** decentralized clinical trial, internet of medical/health things, regulatory science, horizon scanning, citation network, text mining

## Abstract

**Background:**

The need for a new style of clinical trials, called decentralized clinical trials (DCTs), has been increasing as they do not depend on physical visits to clinical sites. DCTs are expected to provide a new opportunity to patients who cannot participate in a clinical trial due to geographical and time limitations. For the adoption of DCTs, it is essential that medical devices with Internet of Medical Things (IoMT) and Internet of Health Things (IoHT) based technologies are developed and commercially adopted. In this study, we aimed to identify the regulatory considerations when IoMT/IoHT-based technologies are used in DCTs or products developed using DCTs.

**Method:**

To understand the study and development field of IoMT/IoHT comprehensively and panoramically, relevant papers published in Web of Science were searched online. Subsequently, a citation network was obtained and characterized as a cluster using a text mining method to identify IoMT/IoHT-based technologies expected to be utilized in DCTs or products developed using DCTs.

**Result and Discussion:**

Upon analysis of the top 15 clusters and subsequent 51 sub-clusters, we identified the therapeutic areas (psychology, neurology) and IoMT/IoHT-based technologies (telemedicine, remote monitoring, and virtual reality) that are expected to be used in DCTs. We also identified several considerations based on the current regulatory guidance.

**Conclusion:**

IoMT/IoHT-based technologies that are expected to be used or products developed using DCTs and key considerations made when they are used in DCTs were identified. The considerations could encourage conducting DCTs using IoMT/IoHT-based technologies.

## Introduction

In the wake of the COVID-19 pandemic, there has been increasing interest in decentralized clinical trials (DCTs) that do not rely on visits to clinical sites to conduct clinical trials. There are two types of DCT-style studies. In one DCT-style, visits to clinical sites can be avoided altogether as several components can be implemented remotely, such as remote electronic informed consent and telemedicine. In the other style (hybrid style), not all study visits are decentralized; certain study visits need to be conducted at the clinical site as usual. For pharmaceutical companies, DCTs reduce subjects' inclusion period and the study population's bias by obtaining study participants from across the globe (1). Prior to the COVID-19 pandemic, DCTs were reported mainly in the United States (US). DCTs are expected to provide new opportunities for participation in clinical trials for patients who have been unable to participate owing to difficulties in accessing clinical sites. They are also expected to reduce the time consumed at the clinical site. According to a patient survey conducted by the Center for Information and Study on Clinical Research Participation (CISCRP), ~60% of patients consider the location of the clinical site and the duration of participation in clinical trials as important factors for study participation (2). Therefore, the location of the clinical site and the duration of the clinical trial are major concerns for patients and, hence, there is room for improvement for study sponsors in these areas. Pharmaceutical companies have been using DCTs to promote patient-centered activities in drug development even before the COVID-19 pandemic.

The advancement of information technology has played a key role in improving the implementation of DCTs. Thus, the adoption of Internet of Things (IoT) in the medical field, in the form of Internet of Medical Things (IoMT) and Internet of Health Things (IoHT), through concepts such as telemedicine, ePRO, and data collection by wearable devices, will enable further dissemination and promotion of DCTs. It is also expected that new IoMT/IoHT-based technologies will be developed while using the components of DCTs to be authorized as qualified medical devices by health authorities such as the FDA. However, considering that the field of IoMT/IoHT has been changing rapidly and the new IoMT/IoHT has been developing constantly, it is difficult to proactively identify the suitable IoMT/IoHT to utilize in DCT and the points to notice when the study sponsor wants to implement the IoMT/IoHT in DCTs.

Therefore, first, we aimed to identify IoMT/IoHT-based technologies that are expected to be utilized in DCTs or products developed using DCTs by using horizon scanning based on bibliographic citation network analysis and text mining. Horizon scanning is a major topic in the strategic initiatives of the International Coalition of Medicines Regulatory Authorities (ICMRA), which is a voluntary coalition of medicines regulatory authorities. According to the concept notes of ICMRA Strategic Priority on Innovation, horizon scanning is broad-reaching information-gathering and monitoring activities to forecast impending products and technologies and potentially disruptive research methods (3).

There are two main types of methods for collecting the data required to conduct well-programmed horizon scanning (4): expert-based and computer-based approaches. Conventional horizon scanning methods have been used in Europe to investigate the Internet, governments, international organizations, companies, databases, and journals using the Delphi method (5, 6). This type of expert-based approach is difficult to implement in the current era of information overload. Computer-based approaches can collect and analyze huge amounts of information and data such as articles, patents, and newspapers. Hines et al. (6) reported that horizon scanning methods are still conducted manually or semi-manually, especially in the medical and healthcare fields, and it is difficult to understand the whole picture of research and technological development because the research results of medical and healthcare fields are extremely large and fragmented. The computer-based approach can supplement the expert-based approach because it is compatible with the scale of the information (7, 8). Two types of computer-based approaches exist: citation and text mining. The citation-based approach, used in this study, assumes that the papers on which a given paper is based are similar to the papers it cites. Thus, it is possible to recognize the whole structure of research areas that comprise a vast volume of papers by analyzing this citation network. Methods based on this approach have been widely used as powerful tools to visualize and understand the structure of research fields and identify new trends and research landscape, and their validity has been identified in various studies (9–11). For example, Kajikawa et al. (12) used citation network analysis to track emerging study areas effectively and efficiently in the field of sustainable science. Similar approaches have been applied in several fields, including energy research (13), regenerative medicine (14), robotics, and gerontology (15). However, it is difficult to correctly understand the semantics of clusters based only on citation relations. Text mining can clarify subject relationships across citations and provide discernment in the dissemination of knowledge in academic research and development. However, it is highly sensitive to certain terms; when text mining is used with other methods, the problem of terminological distortion will be unignorable. Therefore, we propose an objective methodology for horizon scanning that identifies cutting edge technology to be applied to medical products, which are AI-based medical devices, drugs related to T cell immune responses, and extracellular vesicle-based products from all research papers in the target field using both citation network analysis methods and text mining (16–19).

This study aimed to explore novel IoMT/IoHT-based technologies that may be applied in future DCTs using citation network analysis and text mining, both of which are foresight methods used for discovering early signs of emerging products and technologies. In addition, based on the results of citation network analysis and text mining, we aimed to examine the prospects for the utilization of IoMT/IoHT in DCTs and the issues and key considerations that can be assumed to be focused on operational and regulatory perspectives while developing novel IoMT/IoHT-based technologies to contribute to the popularization of IoMT/IoHT in DCTs.

## Method

### Extraction of Paper Data for Analysis

We utilized (“remote” or “virtual” or “Internet” or “wearable” or “sensor”) and (“clinical” or “device”) as queries for analyzing papers published in the Web of Science literature database (WoS, Thomson Reuters) to identify IoMT/IoHT-based technologies that are expected to be used in DCTs and products developed using DCTs.

### Citation Network Analysis

**Figure 1:**
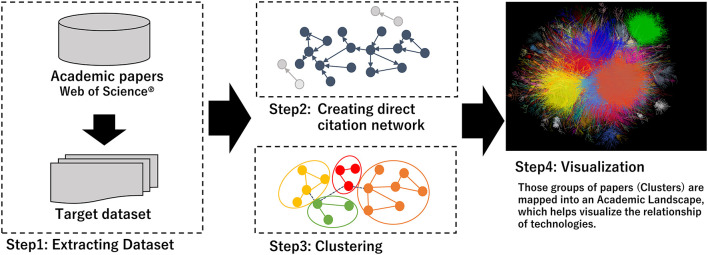
Steps of clustering and making academic landscape.

Figure 1 gives an outline of the citation network analysis process which has been published in references (16, 17, 20). We converted the citation network into an unweighted network, with papers as nodes and citation relationships as links. Papers with no citations as the largest component were considered digressional and excluded from this study (Step 2 in Figure 1). Papers that have no citation relationships with other papers were considered deviant and were excluded from this study. Subsequently, the network was divided into several clusters using the topological clustering method (Step 3 in Figure 1). As a clustering method, topological clustering based on the graph structure of a network is used, and modularity maximization is applied in this study. A cluster module in a citation network is a group of papers in which citation relations are divided using a modularity (Q value) maximization method and are densely aggregated (Louvain method) (20, 21). Q is an evaluation function of the degree of coupling within a cluster and between clusters, as shown in the following equation.


(1) $$\begin{array}{r} Q=\frac{1}{2m}\underset{i,j}{\sum }\left(A_{ij}-\frac{k_{i}k_{j}}{2m}\right)\delta \left(c_{i},c_{j}\right), \end{array}$$

where *A*_*ij*_ represents the weight of the edge between *i* and *j*, $k_{i}=\underset{j}{\sum }A_{ij}$ is the sum of the weights of the edges attached to vertex *i*, *c*_*i*_is the community to which vertex *i* is assigned, δ-function δ(u, v) is 1 if u=v and 0 otherwise, and $m=\;\frac{1}{2}\underset{ij}{\sum }A_{ij}$.

Labels corresponding to the size of the number of papers included were assigned in the clusters. The characteristics of each cluster were confirmed by extracting a summary of frequently cited academic papers and characteristic keywords in the cluster.

Furthermore, we employed term frequency-inverse cluster frequency (TF-ICF) to extract the distinctive keywords of each cluster. TF is a measure of the significance of a term in a particular sentence, whereas ICF is a measure of the general importance of a term. The TC-ICF of a given term *i* in a given cluster *j*, is given by


(2) $$TF-ICF=tf_{i,j}\cdot icf_{i}=tf_{i,j}\cdot \;\log(N/cf_{i})$$

where N is the total number of sentences. Each cluster was labeled based on the resulting keywords and sentences.

The visualization was converted to assume the relationships among these clusters after clustering the network and a large graph layout (LGL) based on a force-direct layout algorithm was used (22, 23). This layout indicated the largest connected component of the network to generate coordinates for nodes in two dimensions. We visualized the citation network by expressing inter-cluster links in the same color (Step 4 in Figure 1). The hub paper, which has the highest number of citations, is located at the center of the citation relations. However, there was no relation of approximation of the content between the position of the clusters and the distance.

### Regulatory Information

Regulatory information for FDA (24), EMA (25), MHLW (26), and PMDA (27) was retrieved from their respective websites.

## Result

### Results of Citation Network Analysis

**Figure 2:**
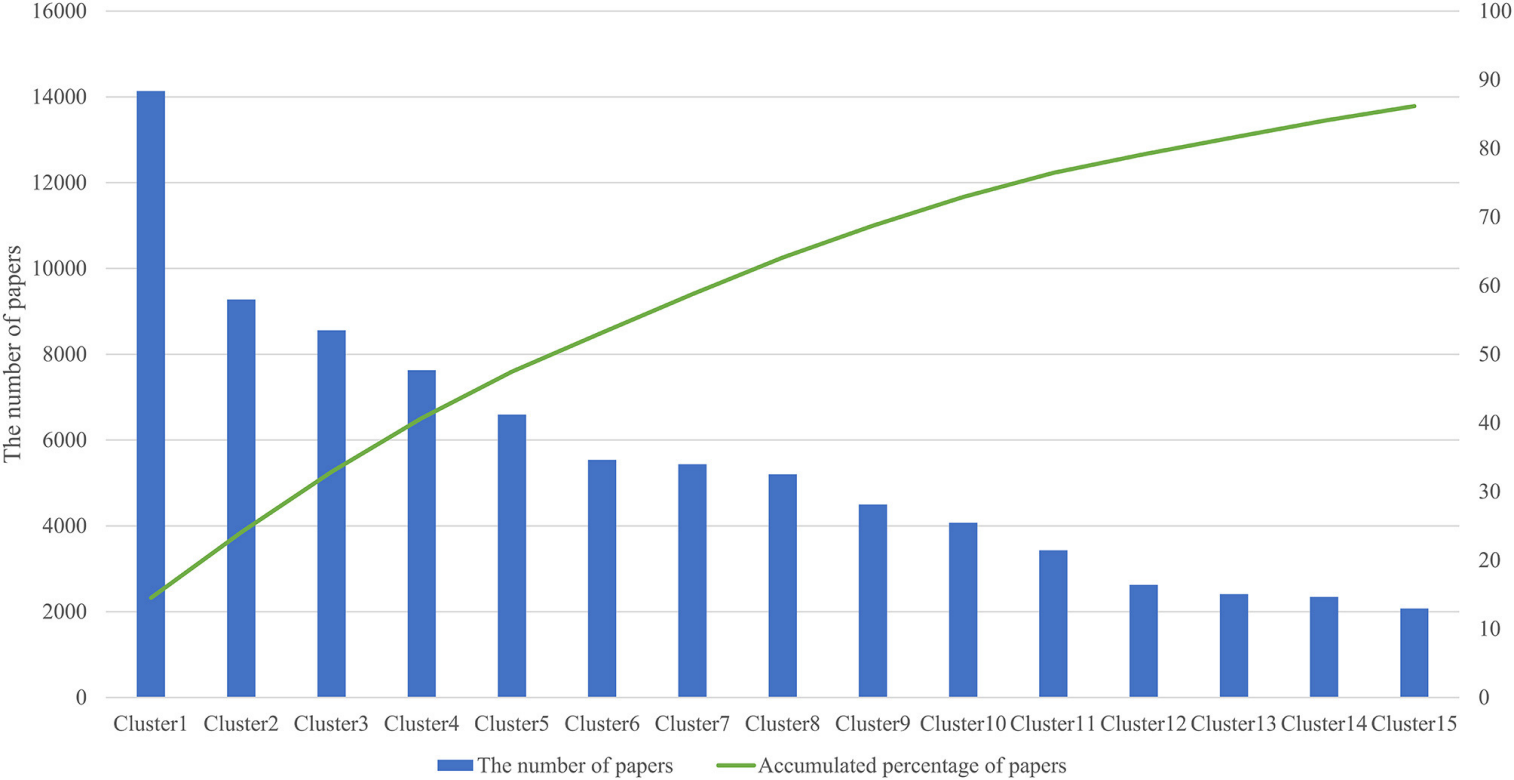
Top 15 cluster size and accumulated percentage.

We analyzed 144,977 papers obtained from the WoS database and discovered that 97,305 papers (67.1%) formed a citation network by extracting the largest connected component from all linkage components through direct citation of papers, and divided it into 141 clusters. Table 1 shows the information of the top 15 clusters (average year, number of papers, TF-ICF, and hub papers in the cluster). Figure 2 shows that the top 15 clusters used for subsequent analysis covered ~90% of the papers in the citation network. The topic of each cluster was assumed in accordance with characteristic keywords with high TF-ICF and the titles and contents of several papers that were most cited within the cluster. For example, cluster 4 was considered to be a group of papers regarding activity-tracking sensors based on the top keywords of the cluster (gait, sleep, and activity). Cluster 6 was categorized as virtual reality in the medical field because the top keywords in the cluster include virtual reality, rehabilitation, and some papers that were cited within the cluster. Cluster 7 was considered a treatment and clinical trial through the Internet with top keywords such as intervention, Internet, trial, and treatment. Cluster 10 was assumed to be associated with remote monitoring mainly focused on telemedicine by referring to the top keywords of the cluster such as telemedicine and the abstract of hub paper of this cluster.

**Table 1:** Information of top 15 clusters for IoM/IoHT.

**Cluster no**.	**Average year**	**Number of papers**	**Top keywords (TF-ICF)**	**Hub paper title**
Cluster 1	2017.6	14134	Iot, thing, network, internet, security, wireless, cloud, smart, computing, authentication	Internet of Things (IoT): A vision, architectural elements, and future directions
Cluster 2	2012.3	9277	Gas, gas sensor, film, sensor, zno, sensing, saw, no2, graphene, gas sensing	Carbon nanotube sensors for gas and organic vapor detection
Cluster 3	2015.0	8559	Electrochemical, electrode, glucose, biosensor, detection, nanoparticles, sensor, biosensors, graphene, microfluidic	Fully integrated wearable sensor arrays for multiplexed *in situ* perspiration analysis
Cluster 4	2016.6	7627	Wearable, gait, physical activity, patient, sleep, walking, activity, wearable device, accelerometer, inertial	A review of wearable sensors and systems with application in rehabilitation
Cluster 5	2017.4	6595	Stretchable, flexible, strain, strain sensor, wearable, graphene, electronics, film, pressure, hydrogel	A stretchable carbon nanotube strain sensor for human-motion detection
Cluster 6	2014.8	5540	Virtual reality, reality, rehabilitation, haptic, virtual, exoskeleton, stroke, patient, limb, motor	Virtual environments for motor rehabilitation: Review
Cluster 7	2013.9	5438	Health, patient, intervention, depression, anxiety, internet, mental health, trial, clinical, treatment	Computer-based psychological treatments for depression: A systematic review and meta-analysis
Cluster 8	2013.5	5204	Fiber, optical, refractive index, refractive, grating, waveguide, plasmon, optical fiber, sensor, surface plasmon	Ultrasensitive photonic crystal fiber refractive index sensor
Cluster 9	2013.5	4502	Student, surgical, skill, surgery, virtual, reality, patient, education, training, virtual reality	Virtual patients: a critical literature review and proposed next steps
Cluster 10	2013.3	4071	Patient, telemedicine, care, heart, defibrillator, heart failure, health, consultation, cardioverter, clinical	Efficacy and safety of automatic remote monitoring for implantable cardioverter-defibrillator follow-up The Lumos-T Safely Reduces Routine Office Device Follow-Up (TRUST) trial
Cluster 11	2016.8	3430	Triboelectric, nanogenerator, teng, harvesting, energy harvesting, piezoelectric, energy, triboelectric nanogenerator, harvester, energy harvester	Transparent triboelectric nanogenerators and self-powered pressure sensors based on micropatterned plastic films
Cluster 12	2018.2	2624	Supercapacitors, flexible, supercapacitor, energy storage, graphene, electrode, wearable, carbon, battery, fiber	Coaxial wet-spun yarn supercapacitors for high-energy density and safe wearable electronics
Cluster 13	2011.9	2409	Mem, sensor, flow, microfluidic, pressure, silicon, resonator, thermal, film, temperature	Micromachined inertial sensors
Cluster 14	2011.5	2342	Magnetic, squid, transition edge, transition edge sensor, magnetic field, superconducting, hall, edge sensor, sensor, magnetometer	Transition-edge sensors
Cluster 15	2013.3	2073	Transistor, field effect, effect transistor, field effect transistor, fet, graphene, gate, biosensor, dna, electrochemical	Multiplexed electrical detection of cancer markers with nanowire sensor arrays

### Characterization of Sub-clusters

Among the top 15 clusters listed in Table 1, we reanalyzed the clusters and distinguished them as sub-clusters that are closely related to the medical and healthcare fields and DCTs. The results are shown in Table 2; 51 sub-clusters are identified. We focused on each sub-cluster in Table 2 to identify the cutting-edge medical devices, technologies, and methodologies that are expected to be used in DCTs or are expected to be developed by DCTs. First, we summarized each sub-cluster as a “summary of sub-cluster” based on keywords, titles, and contents of top-cited papers in the cluster. Subsequently, we identified an example of the device/feature for each sub-cluster. Based on the device/feature example, we identified the therapeutic area and use-case based on information from the sub-cluster summary and the device/feature examples in the sub-cluster. For the therapeutic area in Table 2, we categorized the sub-cluster as “general” when it was not suitable to specify one therapeutic area and/or the device/feature example that may be generally used in the medical field. For the use-case in Table 2, telehealth includes telemedicine, a therapy using IT, such as a digital app for smartphones. Remote monitoring is included in device/feature, which is used for detecting various health information remotely. Table 3 presents the number of therapeutic areas categorized from each sub-cluster. From the results of the characterizations of 51 sub-clusters, 10 are for psychology (20%) and 9 are for orthopedics (18%), which are the main therapeutic areas for utilization of the identified device/feature example from each sub-cluster other than general. For the application of the identified device/feature examples from each sub-cluster, the most common items are telehealth (33%), remote monitoring (27%), rehabilitation (12%), and activity tracking/VR (10%), as shown in Table 4.

**Table 2:** Summarization of sub-cluster focused on DCT-related IoMT/IoHT.

**Cluster no**.	**Average year**	**Number of papers**	**Top keywords (TF-ICF)**	**Summary of sub-cluster**	**Device/feature example**	**Therapeutic area**	**Use-case**
Cluster 1–4	2017.7	1,447	IoT, thing, healthcare, health, cloud, internet, smart, wearable, wireless, network	Issue of IoT for healthcare	Various IoHT-based technologies	General	Remote monitoring
Cluster 2–8	2013.9	472	Breath, nose, exhaled, electronic nose, volatile, exhaled breath, volatile organic, volatile organic compound, gas, organic compound	Evaluation of sensor for a disorder screening	Novel wearable sensor detecting nanomaterials and volatile organic compounds	General	Clinical chemistry
Cluster 3–3	2018	830	Sweat, wearable, glucose, electrochemical, biosensors, electrode, flexible, biosensor, sensor, lactate	Evaluation of wearable sweat sensor	Wearable sensor for sweat analysis	General	Remote monitoring
Cluster 3–4	2017.2	698	Electrochemical, glucose, electrode, graphene, nanoparticles, carbon, h2o2, electrochemical sensor, voltammetry, biosensor	Evaluation of non-enzymic glucose sensor	Non-enzymatic wearable sensor for diabetes	Endocrinology	Remote monitoring
Cluster 3–5	2016.1	588	Wearable, ECG, health, patient, textile, wearable device, QRS, monitoring, heart, wireless	Consideration of wearable monitoring system	Various wearable devices for health monitoring	General	Remote monitoring
Cluster 4–1	2017.8	934	Physical activity, sleep, wearable, activity, fitbit, intervention, health, tracker, activity tracker, physical	Credibility of wearable activity tracker	Activity tracker	General	Activity tracking
Cluster 4–2	2017.2	880	PPG, heart rate, heart, ECG, wearable, photoplethysmography, pulse, atrial fibrillation, atrial, wearable device	Accuracy of smart watch with heart rate monitor	Smart watch with HR monitor	General	Remote monitoring
Cluster 4–3	2016	855	Gait, walking, inertial, gait analysis, wearable, foot, inertial sensor, knee, IMU, gait parameter	Gait analysis with wearable device	Activity tracker	Orthopedics	Activity tracking
Cluster 4–4	2017.1	604	Fall, fall detection, activity recognition, wearable, activity, recognition, accelerometer, human activity, human activity recognition, learning	Analyze of fall detection with wearable device	Activity tracker	Orthopedics	Activity tracking
Cluster 4–5	2017.3	590	Parkinson, gait, tremor, UPDRS, disease, wearable, patient, bradykinesia, motor, dyskinesia	Activity monitoring using wearable sensor for Parkinson's disease patient	Wearable device for Parkinson's disease	Neurology	Activity tracking
Cluster 4–6	2016.8	546	Wearable, intention, wearable device, acceptance, mobile, perceived, learning, adoption, consumer, technology acceptance	Acceptance of wearable technology for health monitoring	Various wearable device for health monitoring	General	Remote monitoring
Cluster 4–7	2016.2	416	Wearable, inertial, rehabilitation, motion, movement, limb, upper limb, exercise, shoulder, stroke	Wearable sensor and system for rehabilitation	Wearable sensor for rehabilitation	Orthopedics	Rehabilitation
Cluster 4–8	2017.2	359	Patient, sleep, radar, vital sign, respiratory, wearable, temperature, COPD, breathing, heart rate	Evaluation of continued monitoring method for vital sign with wearable device	Wearable device with pulse meter	General	Remote monitoring
Cluster 4–10	2014.9	257	Fall risk, fall, gait, older, faller, older adult, frailty, sit, accelerometer, walking	Assessing of fall risk in the elderly using sensors	Wearable inertial sensor	Orthopedics	Activity tracking
Cluster 4–11	2015.8	240	Home, older adult, older, dementia, smart home, smart, cognitive, adult, health, living	Methodology for continued health data collection	Various IoHT-based technologies	General	Remote monitoring
Cluster 4–12	2015.6	238	Wearable, intake, sensecam, physical activity, food, wearable camera, eating, swallowing, dietary, sedentary	Non-invasive monitoring and evaluation of chewing and swallowing	Wearable ingestion monitor	Psychology	Remote monitoring
Cluster 4–14	2012.8	226	Sleep, apnea, sleep apnea, ahi, polysomnography, PSG, PTCCO, hypopnea, oximetry, transcutaneous	Monitoring of a patient with sleep apnea syndrome	Wearable sensor for OSA	Psychology	Remote monitoring
Cluster4–15	2018.4	176	Seizure, EEG, epilepsy, seizure detection, wearable, clonic, ear EEG, ear, clonic seizure, sudep	Evaluation of wearable detector sensor for seizure	Wearable sensor for seizure detection	Neurology	Remote monitoring
Cluster 6–1	2016.2	897	Rehabilitation, stroke, virtual reality, reality, game, motor, balance, upper limb, training, wii	Efficacy of VR therapy for rehabilitation for a stroke	VR	Neurology	VR therapy
Cluster 6–2	2013.3	801	Haptic, force, haptic device, tactile, feedback, haptic interface, force feedback, virtual, robot, haptics	Evaluation of wearable haptic device	Wearable haptic devices	Orthopedics	Rehabilitation
Cluster 6–3	2014.3	604	Virtual reality, reality, anxiety, exposure therapy, phobia, disorder, exposure, virtual, fear, VRET	Evaluation of VR exposure therapy for anxiety	VR	Psychology	VR therapy
Cluster 6–5	2016.9	389	Exoskeleton, walking, gait, robot, limb, wearable, ankle, rehabilitation, lower limb, muscle	Control regulation of lower limb exoskeleton	lower extremity exoskeleton	Orthopedics	Rehabilitation
Cluster 6–6	2015.9	341	Pain, virtual reality, reality, distraction, anxiety, patient, virtual, burn, phantom limb, child	Intervention for pain and burn pain therapy using VR	VR	Neurology	VR therapy
Cluster 6–7	2016.6	320	Exoskeleton, rehabilitation, hand exoskeleton, robot, limb, upper limb, robotic, tremor, wearable, hand	Design and validation of upper limb rehabilitation robot	Robotic prosthetic component	Orthopedics	Rehabilitation
Cluster 6–9	2013.4	224	Telerehabilitation, rehabilitation, patient, telehealth, biofeedback, virtual reality, reality, telepractice, stroke, knee	Evaluation of telerehabilitation with VR	VR	Orthopedics	Rehabilitation
Cluster 6–10	2015.4	219	Virtual reality, reality, cognitive, ADHD, executive, virtual, game, neuropsychological, executive function, concussion	Evaluation of attention deficit using VR	VR	Neurology	VR therapy
Cluster 6–11	2014	190	Prosthesis, myoelectric, limb, EMG, prosthetic, muscle, amputee, mmg, hand, electromyography	Myoelectric control of a prosthetic hand	Robotic prosthetics	Orthopedics	Rehabilitation
Cluster 6–14	2013.7	106	Paranoia, paranoid, delusion, persecutory, psychosis, persecutory delusion, social, schizophrenia, ideation, virtual reality	Efficacy of a psychotic disease therapy with VR	VR	Psychology	VR therapy
Cluster 7–1	2015.8	767	Depression, iCBT, anxiety, treatment, disorder, CBT, intervention, cognitive, mental health, therapy	Depression therapy with internet	Apps for mental health	Psychology	Telehealth
Cluster 7–2	2012.9	566	Health, patient, health information, literacy, internet, website, cancer, information, web, readability	Literacy of health information on the Internet	Various IoHT-based technologies	General	Telehealth
Cluster 7–3	2017.2	491	Mental health, mental, health, depression, apps, intervention, disorder, mobile, mhealth, psychosis	Evaluation of intervention by mobile feature for depression disease	Apps for mental health	Psychology	Telehealth
Cluster 7–4	2014.1	408	Smoking, intervention, cessation, smoking cessation, recruitment, trial, health, participant, alcohol, smoker	Intervention for internet basis smoking abstinence	Apps for smoking abstinence	Psychology	Telehealth
Cluster 7–6	2016	355	Telemedicine, telepsychiatry, patient, telehealth, COVID, mental health, care, clinical, trial, videoconferencing	Study of telemedicine	Telemedicine	General	Telehealth
Cluster 7–7	2012.5	281	Eating disorder, eating, psychotherapy, mental health, social, disorder, mental, CBT, internet, online	Telemedicine for mental healthcare	Telemedicine	Psychology	Telehealth
Cluster 7–9	2015.8	203	Patient, cancer, symptom, decision aid, patient reported, reported outcome, patient reported outcome, outcome, TDCS, care	Evaluation of patient report outcome using internet or mobile app	BYOD ePRO	General	Remote monitoring
Cluster 7–10	2005.9	203	Patient, clinical, decision support, web, medical, internet, wide web, health, world wide web, decision	Evaluation of Electronic Treatment Decision Making System	Telemedicine	General	Telehealth
Cluster 10–1	2014.8	533	Defibrillator, remote monitoring, ICD, patient, cardioverter, cardioverter defibrillator, implantable, implantable cardioverter, implantable cardioverter defibrillator, pacemaker	Evaluation of Efficacy and Safety of Telemonitoring for Follow-up of Implantable Cardioverter Defibrillators	Implantable cardioverter-defibrillator	Cardiology	Life support
Cluster 10–2	2015.2	407	Telemedicine, telehealth, patient, consultation, care, health, service, visit, COVID, clinical	Efficacy of telemedicine	Telemedicine	General	Telehealth
Cluster 10–4	2015.6	378	Heart failure, heart, patient, failure, cardiomems, hospitalization, telemonitoring, remote monitoring, care, pulmonary artery	Remote monitoring for chronic cardiac failure patient	Wireless pulmonary artery hemodynamic monitoring	Cardiology	Remote monitoring
Cluster 10–6	2013.1	275	Patient, diabetes, care, health, portal, patient portal, intervention, mail, provider, telemedicine	Evaluation of an Internet-based Blood Glucose Monitoring System	Telemedicine	Endocrinology	Remote monitoring
Cluster 10–8	2017.2	176	Care, telemedicine, patient, prenatal care, visit, prenatal, pregnancy, telehealth, health, COVID	Telemedicine for infectious disease assessment	Telemedicine	Infectious Diseases	Telehealth
Cluster 10–9	2015.3	175	Glaucoma, ROP, patient, retinopathy, ophthalmologist, optometrist, telemedicine, teleophthalmology, fracture clinic, ophthalmology	Telemedicine in ophthalmology	Telemedicine	Ophthalmology	Telehealth
Cluster 10–10	2012.9	167	Stroke, telemedicine, telestroke, patient, acute stroke, acute, thrombolysis, stroke care, prehospital, care	Accuracy of telemedicine for the stroke	Telemedicine	Neurology	Telehealth
Cluster 10–11	2015.1	156	Patient, telehealth, hypertension, telemedicine, care, telemonitoring, blood pressure, health, home, adherence	Non-invasive remote patient monitoring	Various wearable device for health monitoring	General	Telehealth
Cluster 10–13	2015.6	134	WCD, wearable cardioverter, wearable cardioverter defibrillator, defibrillator, cardioverter, cardioverter defibrillator, ICD, ventricular, patient, sudden cardiac	Evaluation of a wearable defibrillator for arrhythmias with high risk of sudden death	Wearable cardioverter- defibrillator	Cardiology	Life support
Cluster 10–14	2013.3	134	ICU, telemedicine, care, telepharmacy, tele ICU, patient, pharmacy, telehealth, pharmacist, tele	Telemedicine in ICU	Telemedicine	ICU	Telehealth
Cluster 13–4	2015.4	174	Navigation, impaired, blind, impaired people, assistive, sensory substitution, obsacle, travel aid, people, cane	Wearable navigation system for the blind	Electronic travel aids (ETAs), electronic orientation aids (EOAs), and position locator devices (PLDs)	Ophthalmology	Digital health

**Table 3:** Therapeutic area of identified device/feature examples from sub-cluster.

**Therapeutic area**	**No. (%)**
General^a^	16	(28)
Psychology	10	(20)
Orthopedics	9	(18)
Neurology	6	(12)
Cardiology	3	(6)
Ophthalmology	2	(4)
Endocrinology	2	(4)
Otorhinolaryngology	1	(2)
Infectious disease	1	(2)
ICU	1	(2)
Total	51	

a *Represents general content that is not suitable to categorize in one specific therapeutic area*.

**Table 4:** Use-case of identified device/feature examples from sub-clusters.

**Use-case**	**No. (%)**
Telehealth^a^	17	(29)
Remote monitoring^b^	14	(27)
Rehabilitation	6	(12)
Activity tracking	5	(10)
VR therapy	5	(10)
Life support	2	(4)
Clinical chemistry	1	(2)
Digital health^c^	1	(2)
Total	51	

a *Includes telemedicine and therapy using IT, e.g., smartphone app*.

b *A device or feature for detecting health information remotely*.

c *An aggregated condition treated with novel technologies such as AI, VR, and 5G*.

### Regulatory Information for Utilization of IoHT/IoMT in Clinical Trails

Because regulatory matters might be an issue for the utilization of IoMT/IoHT in DCTs, we retrieved relevant regulatory information in the US, EU, and Japan. Table 5 lists the guidelines related to the utilization of IoMT/IoHTs identified in clinical trials. As of February 2022, only Switzerland [Decentralized Clinical Trials (DCTs) with Medicinal Products in Switzerland (30)] and Denmark [Danish Medicines Agency's guidance on the implementation of decentralized elements in clinical trials with medicinal products (31)], have developed guidelines related to DCT. They have included the consideration of how to utilize a relatively proven IoMT/IoHT-based technology, such as a wearable device, in DCTs. In Japan and the US, there are no DCT-specific guidelines. However, there are some guidelines and Q&A documents on the utilization of digital technology (32), such as telemedicine (33), in clinical trials as temporary measures during the COVID-19 pandemic (29, 34–38).

**Table 5:** Guidelines for utilization of IoMT/IoHT in clinical trials or DCT.

**Category**	**Japan (Published year, reference)**	**US (Published year, reference)**	**EU (Published year, reference)**
Tel6medicine and communication devices	MHLW (2019, 28)	FDA (2019, 32)	
	MHLW (2020, 29)	FDA (2019, 33)	
	PMDA (2020, 30)	FDA (2019, 34)	
		FDA (2019, 35)	
Digital technology			EMA (2021, 37)
Data collection process	MHLW (2005, 31)	FDA (2021 draft, 36)	EMA (2021 draft, 38)
			EMA (2010, 39)
DCT			Swissmedic (2021, 40)
			Danish medicines agency (2021, 41)

## Discussion

In this study, we used the horizon scanning method to identify new IoMT/IoHT-based technologies that could be utilized in DCTs or products developed using the DCT approach. Based on the content and keywords (TC-ICFs) of the obtained sub-clusters, we identified technologies and specific medical devices that are pivotal in the citation relationships that make up the sub-clusters. We subsequently categorized them according to therapeutic area. Remote monitoring of biological functions (activity and vitals) using sensors and telemedicine, which are expected to be utilized not only in specific therapeutic areas, was found to be the most common (general). This means that IoMT/IoHT-based technologies, including wearable devices, will be available for use in daily life and clinical trials, including DCTs (1). We discovered that they fit well with therapeutic areas related to mental health, such as psychology and neurology. Telemedicine for psychiatric disorders is already being used in actual clinical practice (39–42), and the COVID-19 pandemic has led to its widespread use (43). In the development of new drugs for psychiatric disorders, conventional clinical trial methods affect the evaluation of drug efficacy because the evaluation by the investigators at each clinical trial institution is subjective and subject to a high degree of variability. To solve this problem, efforts to reduce variability among investigators using telemedicine and centralized evaluation by one principal investigator as a central evaluator have taken center stage. In telehealth, telemedicine is the most commonly used application for remote medical treatment. Devices and technologies aimed at remotely monitoring specific biometrics and disease conditions *via* the Internet were developed. Both telehealth and remote monitoring are important elements of DCTs, and medical devices and infrastructure with these technologies and functions are expected to contribute to the standardization of DCTs. In response to the COVID-19 pandemic, telemedicine and digital health technologies (such as wearable devices) have been applied in the treatment of the COVID-19 to avoid the risk of further hospital-acquired infection. Based on the result of a rapid literature review by Bokolo Anthony Jnr, some considerations have been identified for applying telehealth and digital care solutions during the COVID-19 pandemic (44–46).

We focused on VR therapy as an IoMT/IoHT-based technology that is expected to be utilized and developed through DCTs. VR therapy, as the name suggests, involves the use of VR for medical purposes (47). VR technology enables us to perceive a virtual or artificial world created by a computer as reality. The first VR device was developed by Ivan Sutherland in the US in the 1960s (48, 49); the commercialization of VR devices began in the 1990s, and advancements in the development of VR increased on the brink of the 2000s, with projects such as CAVE (50). In this study, we confirmed that VR therapy has been actively studied in the following disorders and domains: stroke (Cluster 6–1), anxiety (Cluster 6–3), pain therapy (Cluster 6–6), attention deficit (Cluster 6–10), and rehabilitation (Cluster 6–1).

In the US, on November 28, 2021, EaseVRx was authorized by the FDA as a prescription-use immersive VR system that uses cognitive behavioral therapy and other behavioral methods to help with pain reduction in patients aged 18 years and older with diagnosed chronic lower back pain. When used with other treatment methods for chronic lower back pain, this is expected to offer a treatment option for pain reduction that does not include opioid analgesics. In the US, opioid overdose/abuse is a major social problem; therefore, EaseVRx was granted breakthrough device designation. EaseVRx was reviewed through the *de novo* premarket review pathway, a regulatory pathway for low-to-moderate-risk devices of a new type (51). This is the first authorized VR therapy device approved by the FDA as a medical device. In the COVID-19 era, this type of contactless and at-home therapy is more attractive. Because VR therapy can be used remotely at home without visiting an institution and as long as the VR device is with the patient, we believe that it is an appropriate device to be developed at DCTs to conduct clinical trials in an environment where it will be used in actual clinical practice.

### Examples of Novel IoHT/IoMT-based Technologies Expected to Be Developed and Used in DCTs

Based on the information from the sub-cluster in Table 2, we identified potential IoMT/IoHT-based technologies that have not yet been commercialized (Table 6). From Table 6, we assume examples of devices and conceivable use-case scenarios for each device in DCTs. Not all devices are required to obtain approval as medical devices, depending on their application to healthcare workers. Therefore, depending on the product concept of the device, it can be commercialized without regulatory approval; examples include smartwatches and activity trackers. However, to utilize these novel technologies in DCTs as a useful tool, they may be qualified as medical-grade devices by regulatory authorities. The regulatory pathways for many types of devices can be complex. As examples of major regional regulatory authorities in the US, the EU, and Japan, there are some differences in handling medical devices among the three authorities (52–54). Particularly, the EU has unique processes for market access to medical devices in the EU region. Notified bodies that are certified by the EU regulate device approvals. These commercial companies have contracts with device companies to handle device approval, and the Conformité Européenne (CE) mark, which allows the device to be marketed in all EU countries is authorized (55). Most devices are classified based on their level of risk, ranging from class I (lowest risk) to class III (highest risk). This is standardized in the principles of medical devices classification by the Global Harmonization Task Force, which is organized by the US, the EU, Japan, Canada, and Australia (56). The EU has two subclasses in class II: class IIa and class IIb. Class IIa devices are expected to be at a relatively low risk to humans. Class IIb devices are associated with a comparably high risk of harming humans. In the US, the EU, and Japan, class I devices are allowed for market access without any clinical or preclinical data based on their assumed safety and efficacy. However, class II and III devices may require preclinical and safety data verifying their efficacy. The novel IoMT/IoHT-based technologies in Table 6, which are identified based on the citation network, are expected to be classified as higher than class II. Thus, a certain evidence of efficacy may be required for practical use, and the approach of DCT may become a powerful tool for the acceleration and efficiency of the trial because these IoMT/IoHT-based technologies are compatible with remote assessment. As mentioned above, the process for medical device approval and the concept of medical-grade devices differ according to the regulatory authority. These differences by regulatory authorities could become a barrier to the utilization and application of novel IoMT/IoHT-based technologies in DCTs.

**Table 6:** Notable device/features and conceivable use-case in DCTs.

	**Notable device/feature**	**Conceivable use-case in DCTs**
Cluster 3–3	Sweat wearable sensor	Detection of sign of some disease based on change of sweat condition and elements
Cluster 3–4	Non-enzymatic glucose wearable sensor	Remote monitoring of efficacy and safety of diabetic drugs
Cluster 4–12	Automatic ingestion monitor	DCTs for drug development for feeding disorder or obesity

### Key Considerations for the Utilization of IoHT/IoMT in DCTs

It is necessary to consider the appropriateness of using the device with novel technologies that have not yet been in practical use or that have just become commercial realities in clinical trials. Considering the properties of clinical trials, it is desirable to ensure that the quality and accuracy of the data are equal to or higher than that of the data from standardized methods. From the perspective of data quality using IoMT/IoHT in DCTs, it is not mandatory to use a regulatory authorized device as a medical device; however, if it is not, the study sponsor should take measures to ensure data quality in accordance with the accepted principles of clinical trials such as ICH-GCP E6(R2) (57). Guidance from each regulatory authority related to electronic data collection may be useful as a reference to meet the expectations of data collection using IoMT/IoHT-based technologies (28, 58–60). Table 5 shows that guidelines regarding the utilization of IoMT/IoHT in clinical trials are limited. There are certain guidelines for the COVID-19 public health emergency and they have some footholds on how to implement IoMT/IoHT in clinical trials or medical examinations. For example, the digital health device for treating psychiatric disorders is detected in Cluster 7–3 in Table 2, and it is addressed in the FDA policy (29). However, these guidelines for the COVID-19 public health emergency may be effective only during the COVID-19 pandemic; therefore, it is necessary to consider if the permission is temporary. From the experience of the use of digital technology in providing medical care during the COVID-19 pandemic, data privacy and protection has also been identified as a key consideration (46, 61). To secure the data obtained by IoMT/IoHT and promote the utilization of IoMT/IoHT in DCTs, Switzerland and Denmark, with regard to guidance on DCTs from regulatory authorities, are ahead of other countries (30, 31). Because IoMT/IoHT is a key component in conducting DCTs, the considerations for data capturing outside the trial site using mobile technologies are mentioned in the guidelines by Switzerland. IT-equipped medical devices have the potential to completely change the style of clinical trials, which until now have relied on hospital visits and been geographically limited. Therefore, we believe that the active use of new technologies and the accumulation of evidence of utilization in the trial will lead to their secondary use in clinical trials including decentralized-style trials.

### Limitations

We selected WoS as the database for the source of analysis; however, there are other databases, such as PubMed and Scopus, which were excluded. It is necessary to select a database for data source collection that is appropriate for each field of interest. Moreover, there is a time lag between the publication of papers in journals and those reflected in WoS, which causes a time gap in the understanding of the research field. In this study, we focused exclusively on IoMT/IoHT research trends as on the date when the article information was extracted from the database. Additionally, it is necessary to follow the changes in hub papers and other important papers in the research field in chronological order to understand the development of research fields and new technologies. We analyzed the potential IoMT/IoHT-based technologies, disease areas, and purposes of use based on the information in each sub-cluster; however, there is a possibility that subjectivity and bias were incorporated into the evaluation.

## Conclusion

In this study, we investigated the trends related to the adoption of IoT in the medical and healthcare fields (IoMT/IoHT) using citation network analysis and text mining methods. IoMT/IoHT and research fields that are expected to be utilized in DCTs, such as telemedicine, wearable devices, and VR, were identified. Based on the results, we established key considerations in using IoMT/IoHT in DCTs, such as how sponsors can ensure data quality from the IoMT/IoHT-based technology used in DCTs. To the contrary, considering that the field of IoMT/IoHT is evolving rapidly, some relatively recent studies are not included in the dataset used for citation network analysis; they are therefore not identified as hub papers because of having been published quite recently or having few citations. The findings of this study are expected to help in developing the new IoMT/IoHT using DCTs or in using the IoMT/IoHT in DCTs. For future studies, the works of literature and guidance related to DCTs and IoMT/IoHT will be retrieved and examined for continuous solutions to problems based on the latest information and knowledge.

## Data Availability Statement

The original contributions presented in the study are included in the article/supplementary material, further inquiries can be directed to the corresponding author.

## Author Contributions

MS contributed to the conception and design of the study. HS organized the database. HI collected data. HI, TT, and TS performed the data analysis. TS drafted the manuscript. HS and MS reviewed and edited the manuscript. All authors contributed to the article and approved the submitted version.

## Funding

This research was supported by AMED (Japan Agency for Medical Research and Development) Under Grant Number 21mk0101217h0001.

## Conflict of Interest

TS was employed by Astellas Pharma Inc. The remaining authors declare that the research was conducted in the absence of any commercial or financial relationships that could be construed as a potential conflict of interest.

## Publisher's Note

All claims expressed in this article are solely those of the authors and do not necessarily represent those of their affiliated organizations, or those of the publisher, the editors and the reviewers. Any product that may be evaluated in this article, or claim that may be made by its manufacturer, is not guaranteed or endorsed by the publisher.
